# The Development of a Knowledge Test on Transgender Patients’ Care

**DOI:** 10.3390/ijerph17197192

**Published:** 2020-10-01

**Authors:** Jonathan Hernández-Agosto, Kyle Melin, Jurynelliz Rosa-Vega, Edgar Carlo-Frontera, Andrés Rodríguez-Ochoa, Darlene Santiago-Quiñones

**Affiliations:** 1 Curriculum and Institutional Effectiveness Evaluation Division, School of Pharmacy, University of Puerto Rico, San Juan, PR 00936, USA; 2 Department of Pharmacy Practice, School of Pharmacy, University of Puerto Rico, San Juan, PR 00936, USA; kyle.melin@upr.edu (K.M.); jurynelliz.rosa@upr.edu (J.R.-V.); edgar.carlo@upr.edu (E.C.-F.); andres.rodriguez1@upr.edu (A.R.-O.); 3 Department of Pharmacutical Sciences, School of Pharmacy, University of Puerto Rico, San Juan, PR 00936, USA; darlene.santiago@upr.edu

**Keywords:** transgender, transgender care, pharmacy education, continuing education, test validation

## Abstract

The objective of this study was to develop an assessment instrument to measure the effects of a continuing education intervention on 3 domains in pharmacists’ knowledge needed to provide pharmaceutical care for transgender patients: (1) foundations of gender-affirming care, (2) health disparities and the specific needs of transgender patients, and (3) hormone treatments for transgender patients. Multiple-choice questions were developed, and an initial item bank of 47 items was drafted. Item bank revision was conducted by content matter experts, while feedback from 8 practicing pharmacists was provided for face validity and further insights. A preliminary test, containing 42 items was administered to 64 pharmacists before and after a three-hour continuing education intervention. Cronbach’s alpha coefficient yielded a value of 0.65 as a pre-test and 0.77 as a post-test. Items were less difficult to answer by participants after taking the three-hour continuing education, showing better discrimination among high and low performers in the instrument administration as post-test, as well as better correlation when comparing participants’ performance in the overall score against item-level performance. Psychometric evidence supports further instrument examination, which can improve this tool to measure gains in pharmacists’ knowledge related to the care of transgender patients.

## 1. Introduction

One of the biggest challenges researchers often face is how to develop a new instrument, especially when phenomena to be measured is not widely addressed in the literature and the development of a new tailored instrument becomes the best decision. Nonetheless, researchers must consider the relevance of the instrument they want to use to measure any construct or phenomena and place particular interest in research questions as well as the quality of the instrument [1]. In this context, quality can be defined by its validity, “the extent to which an instrument measures what it claims to measure”, and by its reliability, “the extent to which an instrument can be expected to give the same measured outcome when measurements are repeated” [1,2].

Like any other academic field dedicated to the improvement of knowledge by means of research and data gathering, pharmacy education is often at the crossroads of the adoption of existing instruments and the development of new ones to measure health-related phenomena [1]. This communication presents the results of a group of PharmD students and researchers at the University of Puerto Rico School of Pharmacy (SOP) on the development of a new test to measure the effects of a three-hour continuing education (CE) course on the knowledge of pharmacists to provide pharmaceutical care for transgender patients.

It is thought that a lack of knowledge of health care providers may be a contributing factor to the health disparities transgender patients experience when compared to their cis-gender counterparts. In a recent survey of schools of pharmacy, a little over half indicated that transgender-related education is a topic that is currently addressed somewhere within the curriculum, and only 41.2% of schools included it as a required part of the curriculum [3]. Similarly, a 2016 survey of community pharmacy residents found that 72% were not educated about transgender patient issues in pharmacy school and only 36% felt confident in their ability to treat transgender patients [4]. The findings and others demonstrating the limited training of pharmacists in this area have led to several recent calls to incorporate transgender-related care education and training into pharmacy curricula [5,6,7].

In 2017, a study was conducted in Puerto Rico to assess practicing pharmacists’ knowledge and attitudes in providing pharmaceutical care to transgender patients as well as the transgender patients’ perception of pharmacist knowledge and attitude [8]. In this study, pharmacist knowledge was low, with few pharmacists being able to define the term “transgender” or correctly identify appropriate hormonal treatments. Transgender patient perceptions of pharmacist knowledge confirmed these findings, reporting pharmacists to be ill-informed about their healthcare needs. These findings led the University of Puerto Rico SOP to implement a CE course to address these knowledge deficits among practicing pharmacists.

Therefore, it became imperative to evaluate the effectiveness of the CE course to determine if it can contribute to improving pharmacists’ knowledge of the needs of transgender patients as well as their ability to provide pharmaceutical care to these patients. Although transgender patients consistently report low provider knowledge as a barrier to care [9,10,11], we were unable to find an adequate or appropriate existing instrument to measure the cognitive features of pharmacist knowledge. Although several assessments for various healthcare providers have been developed for assessing knowledge of LGBT issues broadly speaking [12,13], most provided very little (or no) assessments specific to transgender care. Many reports regarding healthcare provider knowledge specific to transgender patients have been based on healthcare providers’ self-assessments of their own knowledge [4,14,15]. Ultimately, this resulted in the need to create a new test to assess pharmacist knowledge on providing pharmaceutical care to transgender patients.

We hypothesized that the development of this test may result in a new valid and reliable instrument that allows for the assessment of pharmacists’ knowledge to provide pharmaceutical care to transgender patients. As educators continue to develop educational interventions to address this knowledge deficit among practicing pharmacists, the development of a validated assessment may prove useful in ensuring such interventions are successful in meeting their intended goals. This communication presents the ongoing instrument development procedures and techniques that have been performed to develop an assessment of pharmacists’ knowledge to provide pharmaceutical care to transgender patients. We also describe the capability and quality level of the measurements provided by the instrument at its current phase of development.

The primary goal of this study was to develop a test that enabled researchers to measure the effect of a CE intervention on 3 units in pharmacists’ knowledge needed to provide pharmaceutical care for transgender patients: (1) foundations of gender-affirming care, (2) health disparities and the specific needs of transgender patients, and (3) hormone treatments for transgender patients. Herein, we describe the following steps: the conceptualization of target constructs of knowledge on pharmaceutical care for transgender patients, the development of the content of the initial item pool, the item pool revision and input of technical experts, cognitive interviews with a heterogeneous sample of pharmacists with a similar background to the target educational audience, the final selection of items for the pilot test, and determination of preliminary internal consistency [16,17,18].

## 2. Materials and Methods

### 2.1. Methodology

This section describes the research methodology and the technical aspects for this cross-sectional study and preliminary psychometrical evaluation that were considered and performed in the design, construction, and evaluation of a test to measure pharmacists’ knowledge to provide pharmaceutical care to transgender patients. All subjects gave their informed consent for inclusion before they participated in the study. The study was conducted in accordance with the Declaration of Helsinki, and the protocol was approved by the Institutional Review Board protocol # B0250119 in June 2019, at the Medical Sciences Campus of the University of Puerto Rico.

After confirming that there were no previously published instruments considered adequate or appropriate to address the phenomena intended to be measured, researchers adapted procedures from the three-phase process suggested by Boateng, Neilands, Frongillo, Melgar-Quiñonez, and Young (2018) in their article “Best Practices for Developing and Validating Scales for Health, Social, and Behavioral Research: A Primer to develop a new test” [19]. After a literature review, the first step in the design of this test was the conceptualization of target constructs of knowledge on pharmaceutical care to transgender patients and 3 domains in pharmacists’ knowledge needed to provide pharmaceutical care for transgender patients were identified: (1) foundations of gender-affirming care, (2) health disparities and the specific needs of transgender patients, and (3) hormone treatments for transgender patients. The selection of these domains was based on previous findings from research describing the knowledge and attitudes of pharmacists in Puerto Rico regarding the care of transgender patients [8]. In the previously mentioned 2017 study, a wide variety of knowledge deficits were identified. A lack of understanding of fundamental concepts such as gender identity was evidenced, as well as poor knowledge related to the specific healthcare needs of transgender patients and health disparities they face. Additionally, only 4% and 2% of pharmacists could appropriately identify all hormonal treatments for transgender women and transgender men, respectively. Together, these results were used to inform the 3 domains to be addressed by the continuing education session and assessed by the test to be developed. The second step was the examination of the content to generate the initial pool of items. The current clinical guidelines for the care of transgender patients were used as the primary resource for item development [10,20]. For each of the 3 domains identified, multiple-choice questions were developed, and an initial item bank of 47 items was drafted. The use of multiple-choice items was used to measure the underlying latent knowledge construct as they can additionally account for, and isolate, item-specific measurement error, which can lead to more accurate research findings [16].

The third step entailed the consideration of the content validity to assess if the drafted items were appropriate to measure the 3 domains of pharmacists’ knowledge needed to provide pharmaceutical care for transgender patients. This step was performed by means of a multidisciplinary approach were item bank revision was conducted by content matter experts, as well as other professionals related to the pharmaceutical care for transgender patients with different areas of expertise, that included: (1) pharmaceutical care, (2) curriculum and evaluation, (3) pharmacist professional CE, (4) clinical practice of gender-affirming care, and (5) theoretical frameworks on providing care for sexual and gender minorities. The multidisciplinary team of expert reviewers included practicing clinical pharmacists who provide care for transgender patients, an educational expert on curriculum and evaluation, an educational expert on continuing education for practicing professionals, and a public health expert with extensive experience working with sexual and gender minorities. For the content validity evaluation, the expert reviewers were sent a list of learning objectives for each domain, the 47-item bank, and instructions for completing a semi-structured evaluation which consisted of 6 validity considerations for each item. Reviewers then provided written feedback to the investigators which allowed for any additional feedback or comments they felt was appropriate. Following reviewers’ recommendations, 14 items were modified, and 5 items were dropped, yielding a preliminary test of 42 items. Table 1 shows the item distribution per unit.

The fourth step of the process, after item development and expert judgment, was to perform cognitive interviews with a group of 8 licensed pharmacists, with a similar background and demographic characteristics to the target audience, with the purpose to address possible face validity issues. We wanted to evaluate through the opinion of this group if each item was aligned with the 3 domains, as well as the content relevancy, representativeness, and technical quality [19]. The group of pharmacists offered a variety of suggestions regarding those aspects which were incorporated into the instrument, resulting in an improvement in clarity, syntax, and overall technical quality. Also, they verbalized the mental process they think would be required to provide answers to those questions [19]. A copy of the 42 items Spanish instrument is included in Appendix A. As the fifth step, data collection on items was made in the form of pre- and post-test administrations. While this step does not correspond necessarily to usual validation procedures, as established before, we considered it important to highlight that this test was created primarily to measure pharmacists’ knowledge to provide pharmaceutical care to transgender patients, as a result of a three hour CE activity, which was part of a research project of a group of students of the SOP. The three hour CE was split into 3 sections corresponding with the 3 previously mentioned domains: “Unit 1: Transgender Patient Care Introduction” reviewed basic concepts such as relevant terminology; “Unit 2: General Health Issues of Transgender Patients” covered health disparities and appropriate health screening activities for transgender patients; and “Unit 3: Hormone Replacement Therapy for Gender Affirmation” detailed the pharmacotherapy considerations that are used for gender affirmation.

The preliminary test, containing 42 items, was administered to 64 pharmacists before beginning the CE intervention (pre-test) in the same space as the CE. Participants were recruited when they arrived for the CE presentation and invited to participate in the pilot study of the knowledge test. Participants were first presented with an information sheet explaining their participation was voluntary as well as the study purpose and procedures. The pre-test also collected demographic information including age, sex, gender identity, geographic information, and previous training and experience related to transgender care. Researchers collected all pre-tests before the 3 - h CE session began. The same instrument was administered a second time (post-test) following the CE under identical testing conditions. Participants were given 30 min each to answer the pre- and post-tests. All instruments were pre-printed with matching codes for pre and post-tests to facilitate the paring of the responses in the final analysis.

The sixth step and final step completed and described in this communication consisted of applying statistical and psychometric procedures with the obtained results for instrument preliminary analysis. Measures such as item difficulty index (IDI), item discrimination (ID), item-total correlations (ITC), Cronbach alpha coefficient (α), and confidence intervals for the Cronbach alpha coefficient (CI-α) were computed, to determine initial reference values as well as measurement errors of the instrument. Microsoft Excel (2016), IBM SPSS Statistics for Windows, Version 21.0 (IBM Corporation, Armonk, NY, USA), Intellectus Statistics (Intellectus Statistics LLC, Palm Harbor, FL, USA) and Confidence Intervals (PSYCTC.org, London, England) for a Sample Cronbach alpha Coefficient Value were used for data cleansing, processing, and calculations. Data collection began in August 2019 and finished in November 2019.

### 2.2. Inclusion and Exclusion Criteria

Pharmacists who were (1) licensed to practice in Puerto Rico, (2) members of the Puerto Rico College of Pharmacists (a legal requirement for practice in Puerto Rico), and (3) reported currently practicing pharmacy in Puerto Rico were eligible to participate. No specific exclusion criteria were used aside from excluding those pharmacists who did not meet all three of the inclusion criteria.

### 2.3. Sample Description

All participants were licensed pharmacists practicing in Puerto Rico and attending a 3-h CE session on providing pharmaceutical care for transgender patients. A convenience sample was used where all participants at 2 identical CE offerings (one at the Annual Meeting of the Puerto Rico College of Pharmacists and the other offered at the SOP) could participate in the study. After the CE sessions were completed, data were available for 68 pharmacists, of which 64 completed the pre-test, and 58 completed the post-test. Paired data for both the pre- and post-test was available for a total of 54 pharmacists.

## 3. Results

### 3.1. Demographic Characteristics of the Participants

Most participants 60 (88.2%) identified as heterosexual and cisgender (7 men (10.3%) and 53 women (77.9%)). The remaining participants 4 (11.8%) did not answer that question. A total of 29 (42.6%) participants worked in community pharmacy, 9 (13.2%) in the pharmaceutical industry, 17 (25.1%) in a variety of other scenarios, and 9 (13.2%) did not indicate their practice area. Fifty-three (77.9%) of participants had never received formal training related to transgender care. Only 7 (10.2%) participants indicated they had previously received education on providing care for transgender patients, through CE programs, education as part of the pharmacy curriculum, conferences, or self-study, while 8 (11.8%) did not indicate if they had received previous training in this area.

### 3.2. Assessment of the Preliminary Instrument Results as Pre and Post-Test

A total of 64 pharmacists participated in the administration of the instrument as a pre-test, and 58 participants in the post-test. Table 2 shows the results of the execution scores of each unit. A complete listing of average participant percentage scores for each item can also be found in Appendix B. The total maximum score that a pharmacist could obtain in this test was 1.00 (meaning 100%).

**Figure 1:**
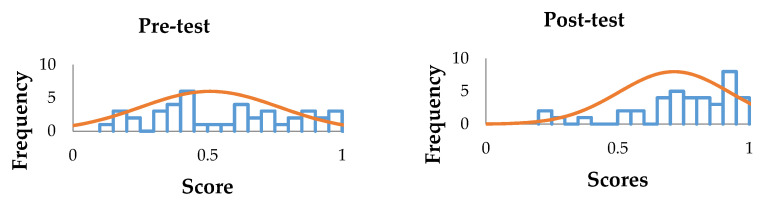
Pre- and post-test variability and spread.

As observed, participants had their highest mean scores in Unit 1, “Introduction to the Care of Transgender Patients” (pre: 0.7299; post: 0.8670). An increase in mean scores also occurred in all units when comparing pre-test and post-test, as well as in the overall results. As shown in Figure 1, the coefficient of variation decreased in average variability or level of dispersion around the mean when comparing pre-test and post-test. As expected, these results show less variation in the post-test modality, since all participants received the same intervention, resulting in a more uniform “understanding” of the concepts being tested.

### 3.3. Basic Psychometrics, Internal Consistency, and Confidence Intervals

Given that we are developing a new measurement instrument, it is important to determine the reliability of its scores, since it has an impact on the precision of the measurement results obtained by it [21]. Table 3 shows a summary of the results for the item difficulty index (IDI), item discrimination (ID), and item-total correlations (ITC). A complete listing IDI, ID, and ITC for each item can also be found in Appendix B.

As shown in Table 3, items were less difficult to answer by participants after taking the three-hour CE. Items also showed better discrimination among high and low performers in the instrument administration as post-test, as well as better correlation when comparing participants’ performance in the overall score against item-level performance.

To review the uniformity of this test, Cronbach’s alpha coefficient (α) analysis was performed to measure the internal consistency of the items in the instrument. The α values obtained for the preliminary instrument when administered as pre-test was α = 0.65 (CI 95%: 0.52–0.76), and when administered as post-test was α = 0.77 (CI 95%: 0.68–0.85). Table 4 shows a summary of the internal consistency and confidence intervals values by mode of administration and unit.

As shown in Table 4, overall internal consistency improved for the instrument when administered as a post-test modality. It also improved for every individual unit in the post-test administration, except for Unit 1.

## 4. Discussion

Here we have presented the preliminary results in our validation process of a new instrument for measuring pharmacists’ knowledge on the topic of providing pharmaceutical care to transgender patients. The instrument was developed because we were unable to find a validated instrument in the literature that adequately measures the cognitive features of pharmacist knowledge of transgender care.

As the results showed, the item difficulty index (IDI) showed an overall improvement, as expected, when comparing pre-test vs. post-test administrations of the instrument. The IDI is the percentage of respondents taking the test who answered the item correctly and where the larger the percentage of getting an item right, the easier the item [22]. An acceptable proportion of the instrument items showed a discrimination index (DI) of 0.20 or more (67% to 81% of the items), as well as item-total correlations (ITC) of 0.20 or more (67% to 86% of the items). Although a DI of 0.20 to 0.29 shows marginal items that may need some revision, this test has its majority over 0.20, while 0.30 and greater are considered good or very good items [23,24]. It is also important to consider what the item intends to measure when analyzing discrimination, which means that looking only at the DI is not sufficient. For example, if the item is intended to measure common basic knowledge or skills that the population to be examined is supposed to master, then both high performers, as well as low performers, should obtain high scores on these kinds of items, which may lower the item discrimination index values, potentially to the point of no discrimination at all.

On the other hand, the ITC was used to find the predictive power of each individual item when compared to the total score. The ITC indicates a positive correlation between respondents’ scores on the item and scores on the test, meaning that those who performed well on that specific item also performed well on the test, and conversely, those who performed poorly on that specific item also performed poorly on the test. Literature suggests that ITC may be useful to provide predictive validity of the total test [22].

When evaluating the Cronbach’s alpha coefficient (α) of the test, these results showed differences among the α with repeated administrations. This is expected, even when it is the same instrument with mostly the same respondents as α values apply to each administration and should not be assumed to be a fixed feature of the scale or instrument [1]. These results compare to what other researchers have found, suggesting the same pattern of α improvement when performing repeated administrations of an instrument to the same population [1,25,26].

To take into consideration the magnitude that α can undergo because of this variations, and due to the influence of the inherent measurement error [23], (especially with a small sample size), confidence intervals for the Cronbach alpha coefficient (CI-α) were computed to improve instrument description in this preliminary assessment, determine the measurement precision of the coefficient for the population, and interpret the level of reliability while accounting for the sampling error in a statistical hypothesis test framework [21]. The CI-α also allows the reader to keep in mind that, as all statistical estimates are affected by sampling error (including reliability), α tests “do not possess absolute or invariant reliability across the samples” [27], particularly with a sample of the aforementioned size, which makes it necessary to calculate its CI-α.

In this particular scenario, when the preliminary instrument was administered as a pre-test, the results showed that for any random sample of size 64 from an infinitely large population in which α = 0.65, the expected α can be expected to be between 0.52–0.76, when calculating the CI-α at 95% certainty for the observed α [28]. On the other hand, when the preliminary instrument was administered as a post-test, the results show that for any random sample of size 58 from an infinitely large population in which α = 0.65, the expected α may be between 0.68–0.85, when calculating the CI-α at 95% certainty for the observed α [28].

It is very common to use distinct attributes, or labels, to describe how appropriate the internal consistency of an instrument is based on the α values. However, there is evidence that suggests a wide and diverse list of terms that can be used to interpret them, supporting that there is no clear consensus on the most appropriate labels to use to describe the values obtained when calculating α [1]. Furthermore, other researchers have suggested that there is no general level (such as 0.70) where α becomes acceptable, but rather that instruments with quite a low value of alpha can still prove been useful in some circumstances [1,29].

With those main considerations in perspective, in this study, the internal consistency coefficients for pre- and post-tests are considered as informative, not final values. However, it is the intention that these values provide a better understanding of instrument capabilities. It is relevant to keep in mind that this test includes specialized items to measure different components of pharmacists’ knowledge to provide pharmaceutical care to transgender patients, which can result in lower α value because of the possible multidimensionality of the items [1].

Another issue that needs to be accounted for when reviewing and interpreting internal consistency is that the sample size, at this point, is still small (*n* = 64). There is literature that suggests at least 300 participants for each scale or an ideal ratio of respondents to items of ten to one (10:1) [19], which means that for this test, at least 300 or a total of 420 respondents may be necessary (42 items × 10 participants each = 420 respondents). Thus far, the test has been administrated to only 64 participants, and increasing the sample size would likely provide a more accurate measure of internal consistency.

With all these aspects already presented, we can certainly affirm that at this point, the overall process has several limitations. First, the results were obtained in a convenience sample of pharmacists who participated in a CE activity and agreed to complete the test. Consequently, this sample is not representative of the entire pharmacist population in Puerto Rico. Second, the sample size requirement is not yet fulfilled. Hence, future steps on continuing the development and validation of this instrument will require an increase in sample size and completion of the validation process by means of doing an Exploratory Factor Analysis (EFA), item reduction process, Confirmatory Factor Analysis (CFA), and test of dimensionality, reliability, and convergent validity.

Despite these limitations, we believe these preliminary results are indicative of an instrument that has the potential to be useful in evaluating pharmacist knowledge needed to provide pharmaceutical care to transgender patients. With the limited incorporation of such content in PharmD curricula today, it is imperative that educational interventions be provided to both PharmD students and practicing pharmacists to improve the collective capacity of the pharmacy profession to adequately care for transgender patients. However, as such educational interventions are developed and implemented, their effectiveness in improving pharmacist knowledge should be assessed. Such assessment is paramount ensuring the quality of such interventions and, ultimately, their ability to improve the care that transgender patients receive. The instrument developed and described here may be of use in evaluating continuing education interventions for practicing pharmacists. Additionally, it may also have the potential to assist in assessing the current knowledge of pharmacists and other healthcare providers in different communities and health systems.

## 5. Conclusions

In this communication, we present a new test that was developed to assess pharmacist knowledge related to pharmaceutical care for transgender patients and the preliminary validation procedures and results for the instrument. The basic psychometric properties of the test are encouraging and demonstrate that the test has the potential to result in a new, valid, and reliable instrument to assess pharmacists’ knowledge on this topic. Preliminary results showed the possibility of measuring changes in pharmacists’ knowledge related to the care of transgender patients, as the results of the pre- and post-tests showed a mean increase in performance of 20.42% (*p* < 0.001), with improvements observed in each of the three units assessed. The instrument has the potential to discriminate between the performance level of respondents, while the Cronbach’s alpha coefficient (α) preliminary analysis described a consistent instrument of measurement. As the profession of pharmacy grapples with the task of improving practicing pharmacists’ knowledge and ability to provide care for transgender patients, the psychometric evidence presented here supports the potential use of this test as a useful tool for measuring the effectiveness of educational interventions in this area. As such, further validation of the instrument is warranted as additional educational interventions for pharmacists are implemented on this topic.

## Figures and Tables

**Table 1:** Item distribution per unit.

Test Unit	Items	Total Items
Unit 1	1–7	7
Unit 2	8–21	14
Unit 3	22–42	21

**Table 2:** Test execution scores by type of administration and unit.

Statistic	Unit 1		Unit 2		Unit 3		Overall Instrument	
	Pre-Test	Post-Test	Pre-Test	Post-Test	Pre-Test	Post-Test	Pre-Test	Post-Test
$\bar{x}$	0.7299	0.8670	0.4944	0.6576	0.4449	0.7011	0.5089	0.7142
*s*	0.216	0.156	0.135	0.144	0.135	0.170	0.1112	0.1251
CV	29.59%	17.99%	27.31%	21.90%	30.34%	24.25%	21.85%	17.52%

$\bar{x}$ = mean; *s* = standard deviation; CV = coefficient of variation.

**Table 3:** Summary of instrument assessment as pre and post-test.

Statistical Test	Pre-Test	Post-Test
IDI	0.08–0.94	0.16–0.98
ID	28 items > 0.20	30 items > 0.20
ITC	28 items > 0.20	36 items > 0.20
	10 items 0 ≥ 0.19	5 items 0 ≥ 0.19
	4 items < 0	1 item < 0

IDI—the item difficulty index; ID—item discrimination; ITC—item-total correlation.

**Table 4:** Internal consistency and confidence intervals by mode of administration and Unit.

Section	α Pre-Test, (95%: CI-α)	α Post-Test, (95%: CI-α)
	*n* = 64	*n* = 58
Overall instrument	0.65; (0.52–0.76)	0.77; (0.68–0.85)
Unit 1	0.54; (0.34–0.69)	0.42; (0.16–0.62)
Unit 2	0.33; (0.06–0.55)	0.43; (0.19–0.62)
Unit 3	0.50; (0.30–0.66)	0.75; (0.65–0.83)

## Appendix B. Items by Unit in Pre and Post-Test

**Unidad**	**Item**	**Pre-Prueba**					**Post-Prueba**				
		**$\bar{x}$**	**SD**	**IDI**	**ID**	**ITC**	**$\bar{x}$**	**SD**	**IDI**	**ID**	**ITC**
**Unidad 1: Introducción al Cuidado de los Pacientes Transgénero**	K 1.1	0.63	0.49	0.63	0.29	0.30	0.98	0.13	0.98	0.06	0.08
	K 1.2	0.89	0.31	0.89	0.24	0.32	0.86	0.35	0.86	0.19	0.21
	K 1.3	0.56	0.50	0.56	0.29	0.31	0.69	0.47	0.69	0.44	0.36
	K 1.4	0.59	0.50	0.59	0.65	0.45	0.79	0.41	0.79	0.25	0.20
	K 1.5	0.59	0.50	0.59	0.76	0.56	0.84	0.37	0.84	0.25	0.26
	K 1.6	0.91	0.29	0.91	0.00	0.07	0.95	0.22	0.95	0.06	0.18
	K 1.7	0.94	0.24	0.94	0.18	0.20	0.95	0.23	0.95	0.19	0.31
**Unidad 2: Otros Problemas de Salud de los Pacientes Transgénero**	K 2.1	0.28	0.45	0.28	0.06	0.05	0.53	0.50	0.53	0.44	0.29
	K 2.2	0.67	0.47	0.67	0.41	0.21	0.79	0.41	0.79	0.13	0.08
	K 2.3	0.08	0.27	0.08	0.12	0.18	0.21	0.41	0.21	0.13	0.02
	K 2.4	0.94	0.24	0.94	−0.06	−0.06	0.88	0.33	0.88	0.13	0.18
	K 2.5	0.66	0.48	0.66	0.47	0.39	0.74	0.44	0.74	0.38	0.38
	K 2.6	0.56	0.50	0.56	0.18	0.19	0.64	0.49	0.64	0.56	0.44
	K 2.7	0.38	0.49	0.38	0.65	0.45	0.66	0.48	0.66	0.38	0.36
	K 2.8	0.38	0.49	0.38	0.59	0.47	0.90	0.31	0.90	0.25	0.32
	K 2.9	0.11	0.31	0.11	−0.12	−0.27	0.53	0.50	0.53	−0.13	−0.05
	K 2.10	0.36	0.48	0.36	0.65	0.51	0.81	0.40	0.81	0.25	0.30
	K 2.11	0.81	0.39	0.81	0.24	0.31	0.62	0.49	0.62	0.44	0.37
	K 2.12	0.14	0.35	0.14	−0.06	−0.09	0.16	0.37	0.16	0.25	0.28
	K 2.13	0.72	0.45	0.72	0.29	0.29	0.86	0.35	0.86	0.38	0.39
	K 2.14	0.84	0.37	0.84	0.12	0.11	0.88	0.33	0.88	0.25	0.30
**Unidad 3: Terapia Hormonal para la Transición de Género**	K 3.1	0.42	0.50	0.42	0.41	0.31	0.62	0.49	0.62	0.25	0.29
	K 3.2	0.52	0.50	0.52	0.41	0.39	0.74	0.44	0.74	0.31	0.41
	K 3.3	0.38	0.49	0.38	0.29	0.26	0.91	0.28	0.91	0.31	0.55
	K 3.4	0.77	0.43	0.77	0.35	0.29	0.91	0.28	0.91	0.25	0.38
	K 3.5	0.45	0.50	0.45	0.29	0.26	0.90	0.31	0.90	0.25	0.37
	K 3.6	0.84	0.37	0.84	0.06	0.08	0.97	0.18	0.97	0.06	0.27
	K 3.7	0.28	0.45	0.28	0.29	0.14	0.33	0.47	0.33	0.44	0.41
	K 3.8	0.34	0.48	0.34	0.35	0.30	0.67	0.47	0.67	0.31	0.37
	K 3.9	0.36	0.48	0.36	0.29	0.23	0.47	0.50	0.47	0.44	0.38
	K 3.10	0.27	0.45	0.27	0.29	0.20	0.84	0.37	0.84	0.13	0.22
	K 3.11	0.34	0.48	0.34	0.24	0.12	0.71	0.46	0.71	0.63	0.44
	K 3.12	0.64	0.48	0.64	0.24	0.24	0.67	0.47	0.67	0.44	0.39
	K 3.13	0.77	0.43	0.77	0.47	0.50	0.88	0.33	0.88	0.19	0.38
	K 3.14	0.31	0.47	0.31	0.29	0.32	0.72	0.45	0.72	0.44	0.43
	K 3.15	0.38	0.49	0.38	0.53	0.42	0.48	0.50	0.48	0.69	0.48
	K 3.16	0.33	0.47	0.33	0.41	0.39	0.76	0.43	0.76	0.50	0.43
	K 3.17	0.66	0.48	0.66	0.29	0.35	0.67	0.47	0.67	0.19	0.25
	K 3.18	0.13	0.33	0.13	0.06	0.13	0.79	0.41	0.79	0.38	0.39
	K 3.19	0.86	0.35	0.86	0.18	0.28	0.86	0.35	0.86	0.31	0.36
	K 3.20	0.16	0.37	0.16	0.00	0.00	0.62	0.49	0.62	0.38	0.30
	K 3.21	0.16	0.37	0.16	−0.06	−0.03	0.19	0.40	0.19	0.38	0.34
$\bar{x}$ = mean score; SD = standard deviation; IDI = item difficulty index; ID = item discrimination; ITC = item-total correlation.											
